# Ethanol Extract of *Piper longum* L., a Culinary Spice, Suppresses Osteoclastogenesis and Protects Against Ovariectomy‐Induced Bone Loss

**DOI:** 10.1111/1750-3841.71232

**Published:** 2026-06-24

**Authors:** Jin‐Ho Moon, Eun‐Young Kim, Sumin Lee, Won Jeong Shin, Seoung Jun Kwon, Youngwoo Nam, Youngjoo Sohn, Hyuk‐Sang Jung

**Affiliations:** ^1^ Department of Anatomy, College of Korean Medicine Kyung Hee University Seoul Republic of Korea

**Keywords:** osteoclastogenesis, ovariectomy‐induced osteoporosis, *Piper longum* L., Piperis Fructus, RANKL signaling

## Abstract

*Piper longum* L., commonly consumed as a culinary spice and referred to as Piperis Fructus (PF) in traditional East Asian medicine, has been associated with various pharmacological activities; however, the anti‐osteoporotic potential of its ethanol extract has not been fully elucidated. In this study, we prepared a PF extract (containing 25.3 mg/g piperine) and investigated its effects on osteoclastogenesis using RANKL‐stimulated RAW 264.7 cells (12.5–50 µg/mL) and ovariectomized (OVX) rats orally administered PF (2.37 and 16.59 mg/kg for 8 weeks). In vitro, anti‐osteoclastogenic effects were evaluated using TRAP, pit formation, F‐actin ring formation, western blot analysis, and qPCR, while in vivo effects were assessed using micro‐computed tomography and serum biomarker analysis. In vitro, PF significantly suppressed osteoclast formation (*p* < 0.05–0.01), TRAP activity (*p* < 0.01), resorption pit area (*p* < 0.01), and F‐actin ring formation (*p* < 0.05–0.01), accompanied by reduced c‐Fos (*p* < 0.01) and NFATc1 protein expression (*p* < 0.05–0.01), as well as the downregulation of osteoclast‐related genes (*p* < 0.05–0.01). In OVX rats, PF‐H significantly reduced serum ALP (*p* < 0.01) and showed a reduction in serum TRAP levels (*p* < 0.05) and improved trabecular microarchitecture with increased BV/TV (*p* < 0.05), Tb.Th (*p* < 0.01), Tb.N (*p* < 0.01), and decreased Tb.Sp (*p* < 0.05). Collectively, PF mitigates estrogen deficiency–induced bone loss by suppressing osteoclast differentiation and function, suggesting its potential relevance for bone health; however, further studies are required.

**Practical Applications:**

*Piper longum* L. is widely consumed as a culinary spice and represents a dietary source of bioactive phytochemicals. The present findings suggest that the ethanol extract of *Piper longum* may help reduce bone loss by suppressing osteoclast activity. These results indicate its potential application as a functional food ingredient or nutraceutical for supporting bone health.

## 1 Introduction

Osteoclast formation and activation are tightly regulated by receptor activator of nuclear factor kappa ligand (RANKL) signaling through its receptor RANK (Feng et al. 2019). The binding of RANKL to RANK on osteoclast precursor cells activates intracellular signaling cascades involving TNF receptor‐associated factor 6 (TRAF6) and key transcription factors, including c‐Fos and nuclear factor of activated T cells cytoplasmic 1 (NFATc1). NFATc1 functions as a central regulator of osteoclastogenesis by inducing osteoclast‐related genes such as cathepsin K (CTK), matrix metalloproteinase‐9 (MMP‐9), osteoclast‐associated receptor (OSCAR), and ATPase H^+^ transporter V0 subunit d2 (ATP6v0d2) (J. H. Kim and Kim 2014). Targeting this pathway is critical for inhibiting bone resorption.

Osteoporosis is a major global health concern characterized by reduced bone mass and microarchitectural deterioration, resulting from an imbalance between osteoclastic resorption and osteoblastic bone formation (Salari et al. 2021). This condition increases fracture risk, particularly in aging populations, and is associated with significant health and socioeconomic burdens. Current pharmacological treatments, including antiresorptive and anabolic agents, are effective but are limited by concerns regarding long‐term safety and adverse effects (B. Kim et al. 2021). These limitations have prompted growing interest in natural products as alternative or complementary approaches.

The dried fruit of *Piper longum* L. (Piperaceae), commonly known as Indian long pepper and referred to as Piperis Fructus (PF) in traditional East Asian medicine, is widely consumed as a culinary spice (Liao et al. 2022). PF has been recognized as a source of bioactive phytochemicals and is increasingly regarded as a dietary component with potential roles in modulating metabolic and inflammatory processes (Kunnumakkara et al. 2018), and it has traditionally been used in East Asian medicine for conditions associated with weakness and chronic disorders (Yu et al. 2020).

Emerging evidence suggests that inflammatory signaling plays an important role in osteoclast differentiation (Kotake et al. 2010), and bioactive constituents of PF, particularly piperine, have been reported to modulate osteoclast activity (Deepak et al. 2015). Previous studies have also shown that aqueous extracts of PF suppress osteoclastogenesis and bone resorption (Gu et al. 2022). However, the effects of ethanol extracts of PF on bone metabolism remain unclear.

Ethanol extraction is known to enrich relatively lipophilic constituents, which may exhibit different pharmacological properties compared with aqueous extracts. Therefore, in the present study, we investigated the anti‐osteoporotic potential of a 100% ethanol extract of PF, focusing on its effects on osteoclast differentiation and bone resorption using RANKL‐stimulated RAW 264.7 cells and an ovariectomized rat model.

## 2 Materials and Methods

### 2.1 Reagents

Recombinant RANKL was used to induce osteoclast differentiation. PF (origin: Indonesia) was procured from Omniherb Co. (Uiseong, Korea). RAW 264.7 murine macrophage cells were obtained from the Korean Cell Line Bank (Seoul, Korea). Estradiol (E2) was used as a positive control for in vivo experiments.

Cell culture media and supplements, including DMEM, α‐MEM, fetal bovine serum (FBS), penicillin/streptomycin, and Dulbecco's phosphate‐buffered saline (DPBS), were purchased from commercial suppliers. The Cell Counting Kit‐8 (CCK‐8), tartrate‐resistant acid phosphatase (TRAP) staining kit, osteo assay plates, and bicinchoninic acid protein assay kit were used for functional and protein assays. Antibodies against c‐Fos, NFATc1, and β‐actin were employed for immunoblot analysis. All other reagents were of analytical grade. Detailed reagent information, including manufacturers and catalog numbers, is provided in Table S1.

### 2.2 Extraction of PF

Dried PF (200 g, Batch No. D1909095159I) was used without grinding and extracted with 100% ethanol (2 L) by maceration at room temperature for 2 weeks. The extract was filtered and concentrated under reduced pressure, followed by lyophilization to obtain the PF ethanol extract (yield: 3.55%). The dried extract was stored at −20°C until use.

### 2.3 Cell Culture and Cell Cytotoxicity of PF

RAW 264.7 cells were maintained in DMEM containing 10% FBS and 1% penicillin/streptomycin at 37°C under 5% CO_2_. For viability assessment, RAW 264.7 cells were plated in 96‐well plates at a density of 5×10^3^ cells per well and incubated for 24 h prior to treatment. Cells were then treated with PF at concentrations of 12.5, 25, and 50 µg/mL either alone or in the presence of RANKL (100 ng/mL) under the same conditions used for osteoclast differentiation and maintained for 5 days. After treatment, 10 µL of CCK‐8 reagent was added to each well, and the plates were further incubated for 2 h. Optical density was determined at 450 nm using a microplate reader. Cell viability was expressed as a percentage relative to untreated controls, and values below 90% were considered cytotoxic. PF was dissolved in DMSO, and the vehicle control group received the same final concentration of DMSO (0.05%) without PF treatment.

### 2.4 Osteoclast Differentiation

RAW 264.7 cells were plated at densities appropriate for each experimental format (5 × 10^3^ cells/well in 96‐well plates, 2 × 10^5^ cells/well in 6‐well plates, and 5 × 10^5^ cells per 60‐mm dish) and incubated for 24 h prior to stimulation. Differentiation was initiated by treatment with RANKL (100 ng/mL) in α‐MEM, either alone or in combination with PF (12.5, 25, or 50 µg/mL). Cells were maintained for 4–5 days to allow osteoclast formation, and the culture medium was refreshed every 2 days.

### 2.5 TRAP Staining and Activity Assay

Differentiated cells were first fixed with 10% formalin for 10 min, followed by washing with phosphate‐buffered saline (PBS). TRAP staining was subsequently carried out using a commercial staining kit according to the supplier's protocol. After drying at room temperature, the stained cells were observed and imaged using a light microscope. Osteoclasts were defined as TRAP‐positive multinucleated cells containing at least three nuclei, and the number of TRAP‐positive cells was quantified for analysis.

For the TRAP enzymatic activity assay, culture media were harvested and centrifuged to eliminate residual debris. The resulting supernatant (50 µL) was incubated with a TRAP reaction mixture composed of p‐nitrophenyl phosphate (pNPP; 4.93 mg), 0.5 M acetate buffer (850 µL), and tartrate solution (150 µL) for 1 h. The reaction was then stopped by adding 0.5 M NaOH, and absorbance was recorded at 405 nm.

### 2.6 Pit Formation Assay

RAW 264.7 cells were cultured on osteo assay surface plates and induced to differentiate into osteoclasts as outlined above. Upon the completion of differentiation, adherent cells were eliminated using 4% NaClO, and the plates were thoroughly rinsed with distilled water. Resorption pits were visualized under an inverted microscope. Resorption pit areas were quantified for analysis.

### 2.7 F‐actin Ring Formation Assay

After osteoclast differentiation, cells were fixed in 4% paraformaldehyde for 20 min and permeabilized with 1% Triton X‐100 in PBS for 5 min. F‐actin was stained with Acti‐stain 488 phalloidin for 30 min at room temperature, followed by nuclear counterstaining with 4′,6‐diamidino‐2‐phenylindole. Fluorescence images were captured at 200× magnification using an immunofluorescence microscope (Selena; Logos Biosystems, MA, USA). F‐actin ring formation was quantified for analysis.

### 2.8 Quantitative Real‐Time Polymerase Chain Reaction

After 4 days of differentiation, total RNA was isolated and quantified using a NanoDrop 2000 spectrophotometer. Two micrograms of RNA were reverse‐transcribed into cDNA using a SuperScript II reverse transcription kit. The synthesized cDNA was amplified with gene‐specific primers. Quantitative real‐time polymerase chain reaction (qPCR) was performed using a CFX Duet Real‐Time PCR System (Bio‐Rad Laboratories, Hercules, CA, USA). The amplification conditions were as follows: initial denaturation at 95°C for 30 s, followed by denaturation at 95°C for 5 s and annealing/extension at 60°C for 10 s. Gene expression levels were normalized to GAPDH as an internal control, and relative expression levels were calculated using the 2^^−ΔΔCt^ method. mRNA expression was assessed at 4 days to reflect the later stages of osteoclast differentiation. Primer sequences are listed in Table S2.

### 2.9 Western Blot Analysis

Following 24 h of differentiation, total cellular proteins were harvested using RIPA lysis buffer containing 50 mM Tris‐Cl, 150 mM NaCl, 1% NP‐40, 0.5% sodium deoxycholate, and 0.1% SDS, supplemented with protease and phosphatase inhibitors. Protein concentrations were quantified using a BCA protein assay. Equivalent amounts of protein were separated by electrophoresis on 10% SDS‐PAGE and subsequently transferred onto nitrocellulose membranes. The membranes were blocked with 5% skim milk for 1 h and then incubated overnight at 4°C with primary antibodies targeting c‐Fos, NFATc1, and β‐actin (1:1000 dilution in 1% bovine serum albumin). After TBST washing, the membranes were treated with HRP‐linked secondary antibodies (1:10,000) for 1 h at room temperature. Protein signals were visualized using an enhanced chemiluminescence detection reagent followed by exposure to X‐ray film. Protein expression of c‐Fos and NFATc1 was analyzed at 24 h, as these are early transcription factors induced during the initial phase of osteoclast differentiation. Detailed information on all antibodies, including sources and catalog numbers, is provided in Table S3.

### 2.10 Induction of Postmenopausal Osteoporosis Model and Treatment

Female Sprague–Dawley rats (11 weeks old, 230–250 g) were housed under controlled environmental conditions (22 ± 2°C, 53%–55% humidity, 12‐h light/dark cycle) with free access to food and water. After a 1‐week acclimatization period, rats underwent bilateral ovariectomy (OVX) under isoflurane anesthesia. Anesthesia was induced with 5% isoflurane and maintained at 3% in 100% oxygen. Sham‐operated animals underwent identical surgical procedures without the removal of the ovaries. Gentamicin (4 mg/kg) was administered for 3 consecutive days after surgery to prevent postoperative infection. Following recovery, animals were randomly assigned to five groups (*n* = 8 per group): Sham‐operated (Sham), ovariectomized (OVX), estradiol‐treated (E_2_), PF low‐dose (PF‐L), and PF high‐dose (PF‐H). The Sham and OVX groups received distilled water as the vehicle. The E2 group was treated with estradiol (100 µg/kg), whereas the PF‐L and PF‐H groups received PF at doses of 2.37 and 16.59 mg/kg, respectively. Estradiol and PF were suspended in distilled water and administered orally once daily for 8 weeks. Following the experimental period, the rats were euthanized, and blood, uterus, and femur samples were collected. All animal procedures were conducted in accordance with the guidelines approved by the Institutional Animal Care and Use Committee of Kyung Hee University (KHSASP‐21‐185). The dosing regimen for PF was determined based on traditional herbal usage and extraction yield. PF is prescribed at approximately 4 g/day for a 60‐kg adult according to classical herbal medicine references (Korean Oriental Medical Colleges 2020). Considering an extraction yield of 3.55%, this corresponds to approximately 142 mg/day of extract, equivalent to 2.37 mg/kg, which was selected as the low dose based on the human daily intake expressed on a body weight basis. To assess dose‐dependent pharmacological effects, a higher dose (16.59 mg/kg), approximately sevenfold higher than the low dose, was included to provide a broader pharmacological exposure range in the preclinical model. Based on the quantified piperine content of 25.3 mg/g extract, the estimated piperine exposure corresponds to approximately 0.060  and 0.420 mg/kg for the PF‐L and PF‐H groups, respectively.

### 2.11 Serum Analysis

Serum TRAP activity was assessed using a pNPP‐based assay. The procedure was performed under conditions comparable to those used for the cellular TRAP activity assay. Serum alkaline phosphatase (ALP) levels were measured by DK Korea (Seoul, Korea).

### 2.12 Micro‐Computed Tomography

Femurs were scanned using a SkyScan1176 micro‐computed tomography (micro‐CT) system (Bruker, Kontich, Belgium). The scanning conditions were set at 50 kV and 200 µA with a voxel resolution of 8.9 µm and a 0.5‐mm aluminum filter. Image acquisition was conducted with a rotation step of 0.4° across a total angle of 180°. The acquired projection images were reconstructed using the NRecon software (version 1.6.10.1; Bruker Corporation, MA, USA) with standard correction for beam hardening and ring artifacts. The region of interest was selected in the distal femur, starting 1 mm proximal to the growth plate and extending proximally for an additional 1 mm. A global threshold was applied to segment mineralized tissue, and identical threshold settings were consistently used for all samples. Trabecular microstructural indices, including BV/TV, Tb.Th, Tb.N, and Tb.Sp, were quantified using the CTAn software (Bruker Corporation).

### 2.13 LC‐MS Analysis

PF samples were analyzed by LC–MS using an Alliance E2695 HPLC system coupled with an ACQUITY QDA mass detector (Waters, MA, USA). Chromatographic separation was performed on an XBridge C18 column (250 × 4.6 mm, 5 µm) at room temperature with a flow rate of 0.3 mL/min. The mobile phase consisted of 0.1% formic acid in water (solvent A) and acetonitrile (solvent B). A gradient elution program was applied in which solvent B was increased from 0% to 99% over 40 min, reduced to 75% between 40 and 45 min, and subsequently raised again to 99% from 45 to 50 min. The injection volume was set to 10 µL. Mass spectrometric detection was conducted in positive electrospray ionization mode with a capillary voltage of 0.8 kV, a cone voltage of 15 V, and a scan range of m/z 100–600. Piperine was identified as the principal marker compound based on comparison of its retention time with that of an authentic standard. The quantification of piperine in the PF extract was performed using an external standard calibration curve.

### 2.14 Statistical Analysis

All in vitro data represent results from at least three independent experiments and are expressed as mean ± SEM. Statistical significance was determined using one‐way ANOVA followed by Dunnett's post hoc test. For in vitro analyses, each independent experiment was considered a biological replicate, and technical replicates were averaged prior to analysis. For longitudinal body weight data (Figure 5A), a two‐way repeated‐measures ANOVA followed by Sidak's multiple comparisons test was used. All analyses were performed using GraphPad Prism (version 9; GraphPad Software Inc., CA, USA), with *p* < 0.05 considered statistically significant. The ImageJ software (National Institutes of Health, MD, USA) was used for the quantification of F‐actin rings, resorption areas, and densitometric analysis.

## 3 Results

### 3.1 Effects of PF on Osteoclast Formation and Cell Viability

**FIGURE 1:**
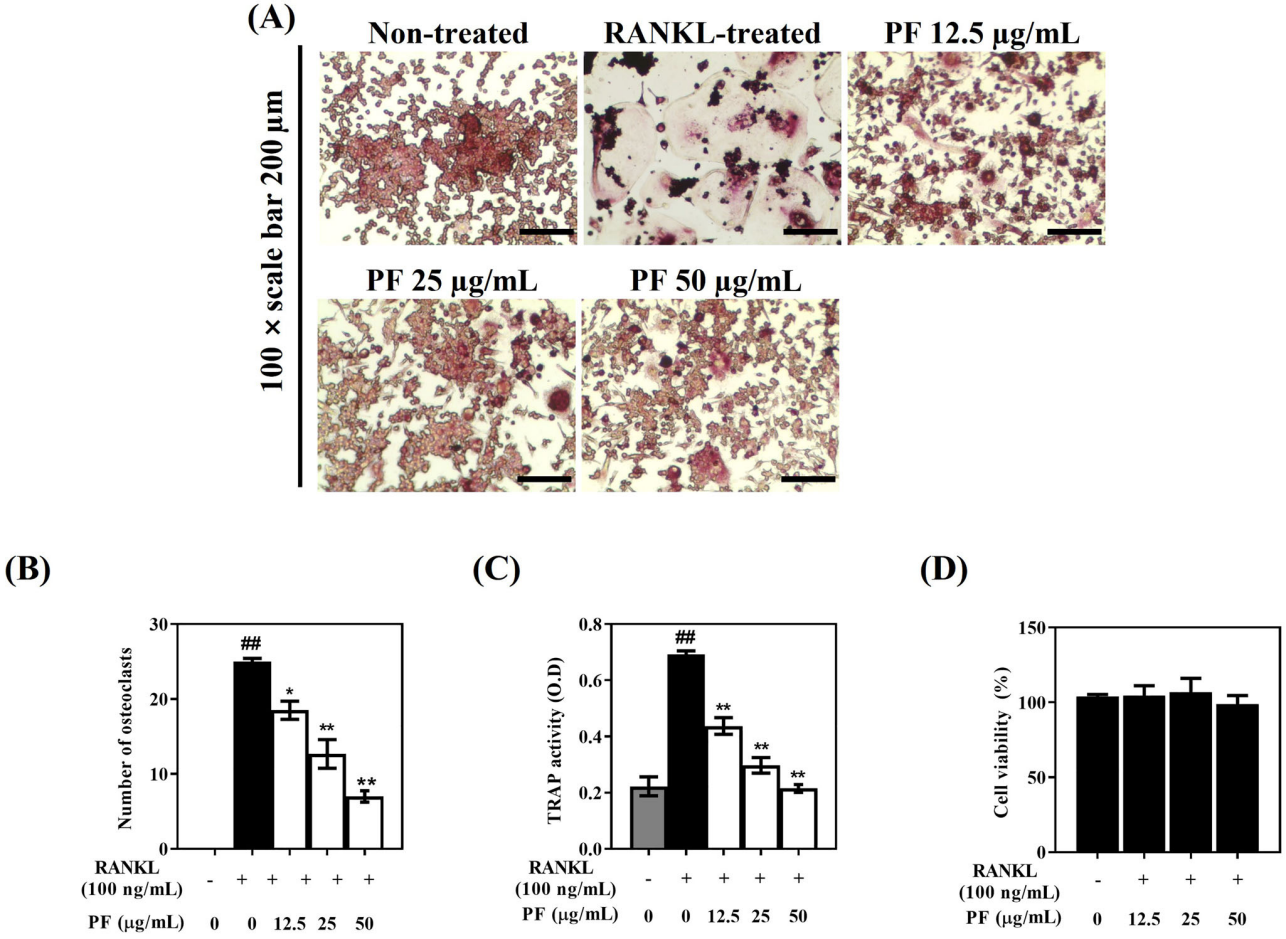
Effects of Piperis Fructus (PF) on osteoclast formation. Osteoclast differentiation was evaluated by tartrate‐resistant acid phosphatase (TRAP) staining following treatment with PF (12.5, 25, and 50 µg/mL) in the presence of receptor activator of nuclear factor‐κB ligand (RANKL, 100 ng/mL). (A) Representative images were obtained using light microscopy. (B) TRAP‐positive multinucleated cells containing three or more nuclei were counted. (C) TRAP activity in the culture medium was determined using a p‐nitrophenyl phosphate‐based assay. (D) Cell viability of RAW 264.7 cells treated with PF (12.5, 25, and 50 µg/mL) in the presence of RANKL (100 ng/mL) under the same osteoclast differentiation conditions for 5 days. Data are presented as mean ± SEM (n = 3 independent experiments). #*p* < 0.05 and ##*p* < 0.01 versus untreated cells; *p < 0.05 and **p < 0.01 versus RANKL‐treated cells.

Upon RANKL stimulation, RAW 264.7 cells differentiated into multinucleated osteoclasts that were strongly positive for TRAP staining (Figure 1A). Quantitative analysis demonstrated that osteoclast formation was significantly increased by RANKL treatment compared with non‐treated cells (*p* < 0.01) (Figure 1B). Treatment with PF markedly suppressed osteoclast differentiation, with significant inhibition observed at 12.5 µg/mL (*p* < 0.05) and at 25 and 50 µg/mL (*p* < 0.01 for both) compared with the RANKL‐treated group (Figure 1A,B). Consistent with these findings, TRAP activity in the culture medium was significantly reduced at 12.5, 25, and 50 µg/mL compared with the RANKL‐treated group (*p* < 0.01 for all) (Figure 1C). In addition, to exclude the possibility that the inhibitory effects of PF were due to cytotoxicity, cell viability was assessed under the same differentiation conditions. PF treatment did not significantly affect the viability of RAW 264.7 cells under the same differentiation conditions over the 5‐day period, with cell viability remaining above 90% across all tested concentrations (Figure 1D).

### 3.2 Effects of PF on Pit Formation and F‐Actin Ring Formation

**FIGURE 2:**
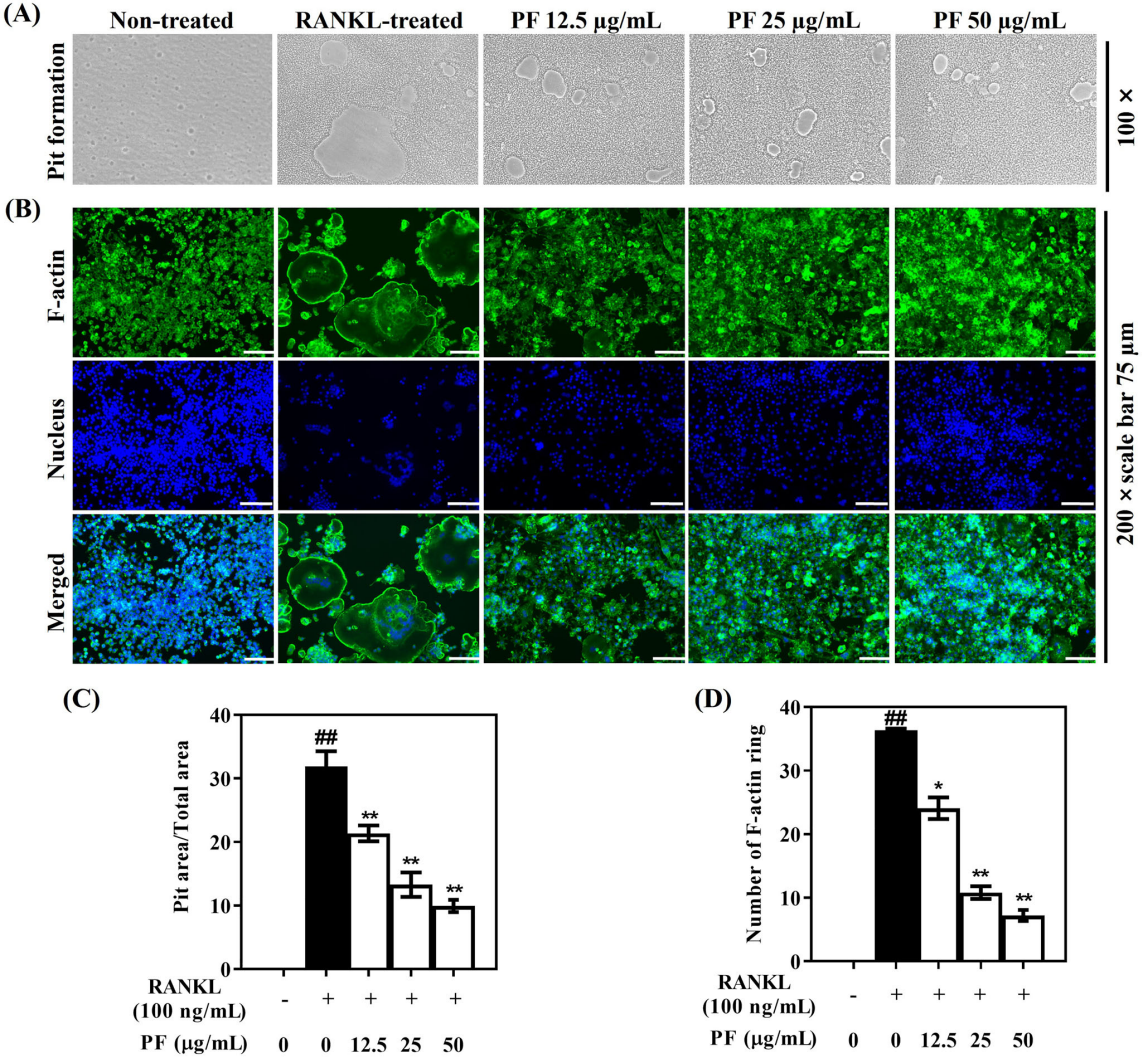
Effects of Piperis Fructus (PF) on pit formation and F‐actin ring formation in osteoclasts. (A) Resorption pits formed on osteo assay plates were observed using inverted microscopy following treatment with PF (12.5, 25, and 50 µg/mL) in the presence of receptor activator of nuclear factor‐κB ligand (RANKL, 100 ng/mL). (B) F‐actin ring structures were visualized by fluorescence staining; representative images show F‐actin (green) and nuclei (blue). (C) Resorption pit area and (D) the number of F‐actin rings were quantified. Data are presented as mean ± SEM (*n* = 3 independent experiments). #*p* < 0.05 and ##*p* < 0.01 versus untreated cells; *p < 0.05 and **p < 0.01 versus RANKL‐treated cells.

RANKL stimulation markedly increased bone resorption pit formation compared with non‐treated cells, whereas PF treatment suppressed pit formation (Figure 2A). Quantitative analysis demonstrated that PF dose‐dependently reduced the resorption pit area compared with the RANKL‐treated group (*p* < 0.01 for all PF concentrations) (Figure 2C). RANKL treatment induced the formation of large and well‐organized F‐actin rings, accompanied by multinucleated mature osteoclast morphology, whereas PF treatment disrupted F‐actin ring formation and reduced multinucleated osteoclast morphology (Figure 2B). Quantitative analysis showed that PF dose‐dependently reduced F‐actin ring formation, with significant reductions observed at 12.5 µg/mL (*p* < 0.05) and at 25 and 50 µg/mL (*p* < 0.01 for both) compared with the RANKL‐treated group (Figure 2D).

### 3.3 Effects of PF on c‐Fos and NFATc1 Protein Expression

**FIGURE 3:**
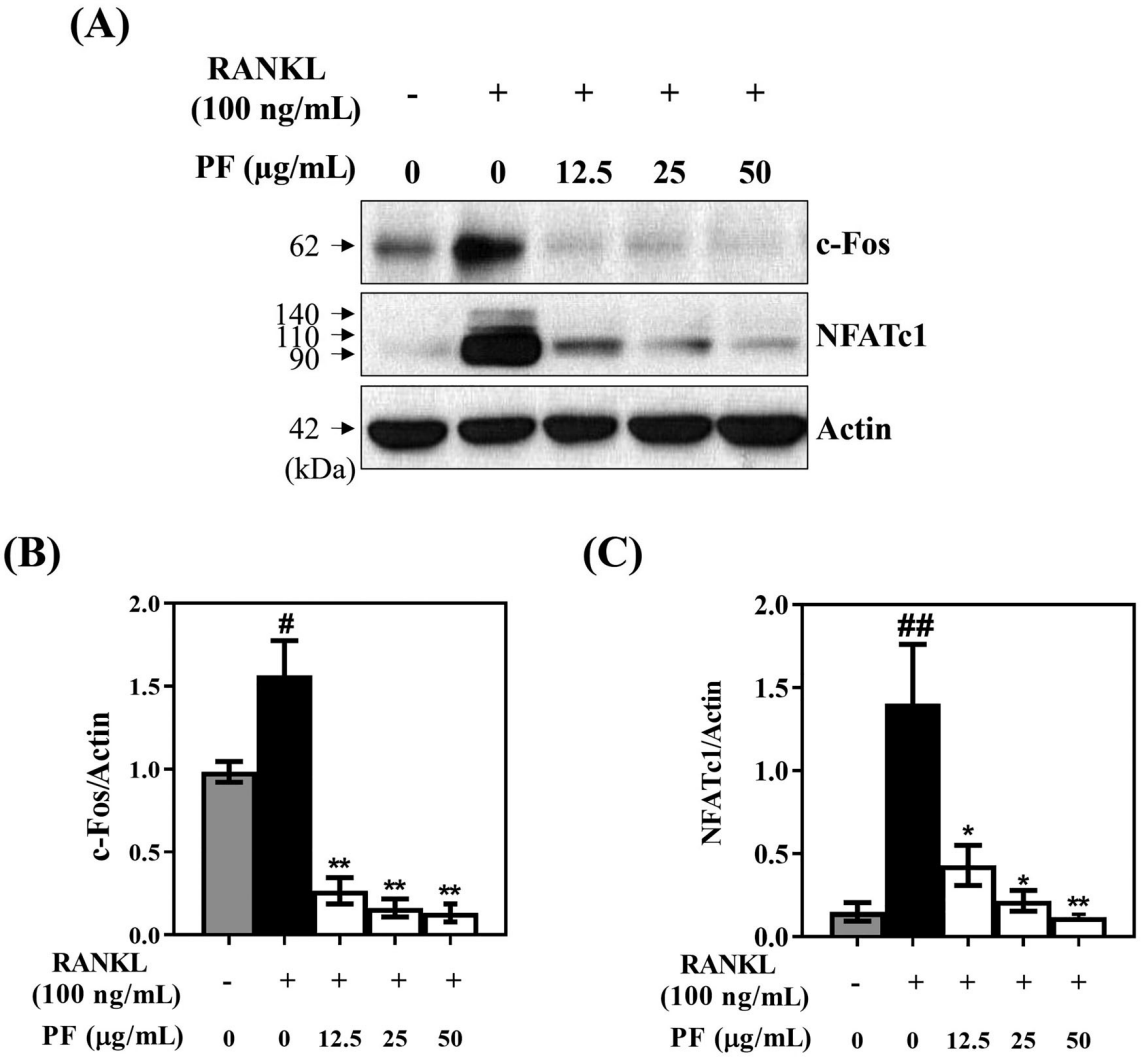
Effects of Piperis Fructus (PF) on c‐Fos and nuclear factor of activated T‐cells, cytoplasmic 1 (NFATc1) protein expression. (A) Representative western blot images showing c‐Fos and NFATc1 protein levels following treatment with PF (12.5, 25, and 50 µg/mL) in the presence of receptor activator of nuclear factor‐κB ligand (RANKL, 100 ng/mL). (B, C) Quantitative analysis of c‐Fos and NFATc1 band intensities normalized to β‐actin. Data are presented as mean ± SEM (*n* = 3 independent experiments). #*p* < 0.05 and ##*p* < 0.01 versus untreated cells; *p < 0.05 and **p < 0.01 versus RANKL‐treated cells.

Stimulation with RANKL markedly elevated the expression levels of c‐Fos and NFATc1 proteins compared with non‐treated cells (Figure 3A). The administration of PF suppressed this RANKL‐driven increase in protein expression. Densitometric analysis of the western blot bands, normalized to β‐actin, demonstrated that PF treatment significantly lowered c‐Fos protein abundance at 12.5, 25, and 50 µg/mL (*p* < 0.01 for all) compared with the RANKL‐treated group (Figure 3B). NFATc1 protein abundance was also significantly reduced by PF treatment at 12.5 µg/mL (*p* < 0.05) and at 25 and 50 µg/mL (*p* < 0.01 for both) compared with the RANKL‐treated group (Figure 3C).

### 3.4 Effects of PF on Osteoclast‐Related Gene Expression

**FIGURE 4:**
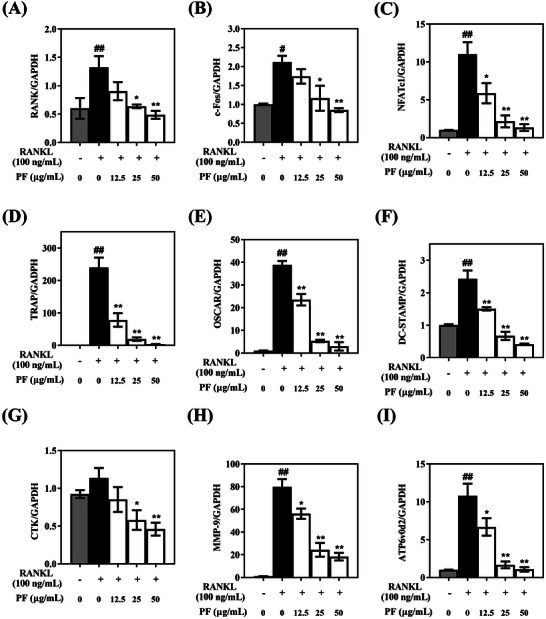
Effects of Piperis Fructus (PF) on osteoclast‐related gene expression assessed by quantitative real‐time polymerase chain reaction (qPCR). Relative mRNA expression levels of osteoclast‐related genes were analyzed by qPCR after treatment with PF (12.5, 25, and 50 µg/mL) in the presence of receptor activator of nuclear factor‐κB ligand (RANKL, 100 ng/mL) for 4 days. (A) Receptor activator of nuclear factor‐κB (RANK), (B) c‐Fos, (C) nuclear factor of activated T‐cells cytoplasmic 1 (NFATc1), (D) tartrate‐resistant acid phosphatase (TRAP), (E) osteoclast‐associated receptor (OSCAR), (F) dendritic cell‐specific transmembrane protein (DC‐STAMP), (G) cathepsin K (CTK), (H) matrix metalloproteinase‐9 (MMP‐9), and (I) ATPase H+ transporting V0 subunit d2 (ATP6v0d2). Data are presented as mean ± SEM (*n* = 3 independent experiments). Statistical significance was indicated by #*p* < 0.05 and ##*p* < 0.01 versus untreated controls and *p < 0.05 and **p < 0.01 versus RANKL‐treated cells.

Quantitative real‐time PCR analysis showed that RANKL stimulation markedly elevated the mRNA expression levels of genes associated with the RANK/c‐Fos/NFATc1 signaling axis, osteoclast differentiation markers, and osteoclast fusion‐ and bone resorption‐related genes (Figure 4A–I). RANK and c‐Fos expression were significantly reduced by PF at 25 µg/mL (*p* < 0.05) and 50 µg/mL (*p* < 0.01) compared with the RANKL‐treated group (Figure 4A,B). NFATc1 expression was significantly reduced at 12.5 µg/mL (*p* < 0.05) and at 25 and 50 µg/mL (*p* < 0.01 for both) compared with the RANKL‐treated group (Figure 4C). TRAP, OSCAR, and DC‐STAMP expression were significantly reduced by PF at 12.5, 25, and 50 µg/mL (*p* < 0.01 for all) compared with the RANKL‐treated group (Figure 4D–F). Among the bone resorption‐related genes, CTK expression was significantly decreased at 25 µg/mL (*p* < 0.05) and 50 µg/mL (*p* < 0.01), whereas MMP‐9 and ATP6v0d2 expression were significantly reduced at 12.5 µg/mL (*p* < 0.05) and at 25 and 50 µg/mL (*p* < 0.01 for both) compared with the RANKL‐treated group (Figure 4G–I).

### 3.5 Effects of PF on Body and Organ Weights and Serum Biomarkers in OVX Rats

**FIGURE 5:**
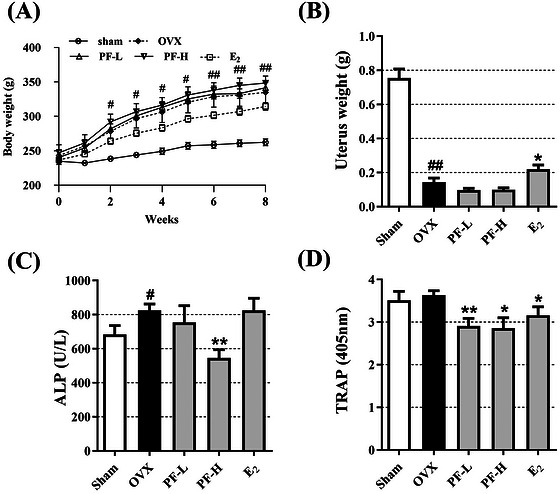
Effects of Piperis Fructus (PF) on body weight, organ weights, and serum biomarkers in ovariectomized (OVX) rats. OVX rats were orally administered PF low‐dose (PF‐L, 2.37 mg/kg/day) and PF high‐dose (PF‐H, 16.59 mg/kg/day). (A) Changes in body weight over the experimental period. (B) Uterine weight. (C) Serum alkaline phosphatase (ALP) levels. (D) Serum tartrate‐resistant acid phosphatase (TRAP) levels. Each group consisted of eight animals. Data are presented as mean ± SEM (*n* = 8 per group). Statistical significance was indicated by #*p* < 0.05 and ##*p* < 0.01 versus the Sham‐operated (Sham) group and *p < 0.05 and **p < 0.01 versus the OVX group.

Ovariectomy resulted in progressive body weight gain compared with the Sham group. PF‐L (2.37 mg/kg) and PF‐H (16.59 mg/kg) did not significantly alter body weight relative to the OVX group (Figure 5A).

Uterine weight was markedly decreased in OVX rats compared with the Sham group (*p* < 0.01), whereas E_2_ treatment significantly increased uterine weight relative to the OVX group (*p* < 0.05). PF‐L and PF‐H did not significantly influence uterine weight (Figure 5B). To assess bone turnover status, serum biochemical markers were evaluated. OVX significantly elevated serum ALP levels compared with the Sham group (*p* < 0.05), whereas PF‐H (16.59 mg/kg) significantly attenuated this increase relative to the OVX group (*p* < 0.01) (Figure 5C). In contrast, serum TRAP levels were not significantly different between the Sham and OVX groups; however, PF‐L (2.37 mg/kg) significantly reduced TRAP levels relative to the OVX group (*p* < 0.01), and both PF‐H (16.59 mg/kg) and E_2_ also significantly decreased TRAP levels compared with the OVX group (*p* < 0.05) (Figure 5D).

### 3.6 Effects of PF on Bone Microarchitecture

**FIGURE 6:**
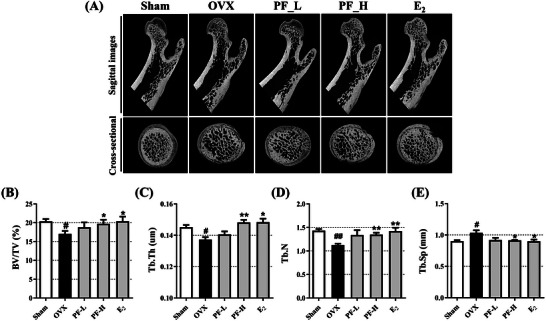
Effects of Piperis Fructus (PF) on trabecular bone microarchitecture in ovariectomized (OVX) rats. OVX rats were orally administered PF low‐dose (PF‐L, 2.37 mg/kg/day) and PF high‐dose (PF‐H, 16.59 mg/kg/day). (A) Representative micro‐computed tomography (micro‐CT) images of distal femoral trabecular bone. (B) Bone volume/total volume (BV/TV), (C) trabecular thickness (Tb.Th), (D) trabecular number (Tb.N), and (E) trabecular separation (Tb.Sp). Quantitative analysis was performed to assess structural changes following PF treatment. Data are expressed as mean ± SEM (*n* = 8 per group). Statistical significance was indicated by #*p* < 0.05 and ##*p* < 0.01 versus the Sham‐operated (Sham) group and *p < 0.05 and **p < 0.01 versus the OVX group.

Micro‐CT evaluation revealed marked deterioration of trabecular structure in OVX rats compared with the Sham group (Figure 6A). BV/TV was significantly reduced in OVX rats compared with the Sham group (*p* < 0.05), whereas PF‐H (16.59 mg/kg) and E_2_ significantly increased BV/TV relative to the OVX group (both *p* < 0.05) (Figure 6B). Tb.Th was significantly reduced in OVX rats compared with the Sham group (*p* < 0.05), while PF‐H (16.59 mg/kg) and E_2_ significantly increased Tb.Th relative to the OVX group (*p* < 0.01 and *p* < 0.05, respectively) (Figure 6C). Tb.N was significantly reduced in OVX rats compared with the Sham group (*p* < 0.01), whereas PF‐H (16.59 mg/kg) and E_2_ significantly increased Tb.N relative to the OVX group (both *p* < 0.01) (Figure 6D). In contrast, Tb.Sp was significantly increased in OVX rats compared with the Sham group (*p* < 0.05), while PF‐H (16.59 mg/kg) and E_2_ significantly decreased Tb.Sp relative to the OVX group (both *p* < 0.05) (Figure 6E).

### 3.7 LC‐MS Analysis of PF

**FIGURE 7:**
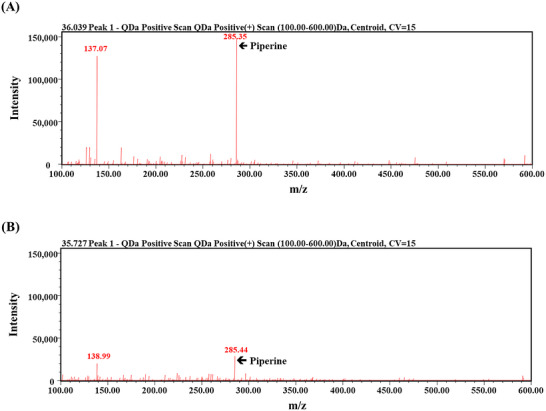
Liquid chromatography‐mass spectrometry (LC‐MS) of Piperis Fructus (PF) extract and piperine standard. (A) LC‐MS spectrum of the piperine standard showing the characteristic molecular ion peak at m/z 285.35. (B) LC‐MS spectrum of the PF extract showing the corresponding molecular ion peak at m/z 285.44. Positive ion electrospray ionization (ESI+) mode was used with a scanning range of m/z 100–600. The arrows indicate the piperine peaks.

Piperine was identified as a representative marker compound within the extract. Quantitative analysis revealed that the PF extract contained 25.3 mg/g of piperine. In the standard spectrum (Figure 7A), piperine exhibited a prominent peak at m/z 285.35 in positive ion mode. Similarly, in the PF extract spectrum (Figure 7B), a corresponding peak was observed at m/z 285.44, confirming the presence of piperine in the extract. These results confirm the presence of piperine in the PF extract. Based on this quantification, the estimated piperine exposure corresponding to the administered PF doses ranged from 0.060 to 0.420 mg/kg.

## 4 Discussion

Previous studies have reported the anti‐osteoporotic potential of PF, primarily using aqueous extracts and focusing on bone‐protective effects (Gu et al. 2022). In contrast to those studies, the present work evaluated a 100% ethanol extract, focusing specifically on osteoclast differentiation and bone resorption in both a RANKL‐stimulated RAW 264.7 model and an ovariectomized (OVX) rat model. PF markedly reduced osteoclast formation and resorptive function in vitro, as evidenced by decreased TRAP staining, reduced pit formation, and impaired F‐actin ring organization, along with the downregulation of osteoclast‐related genes. These cellular effects were paralleled in vivo, where PF administration preserved trabecular microarchitecture and mitigated osteoclast‐driven bone turnover in OVX‐induced osteoporosis. Given that PF is traditionally consumed as a culinary spice, these findings may provide insight into the potential role of spice‐derived dietary bioactive compounds in modulating bone metabolism through regular dietary intake.

TRAP is commonly used to evaluate osteoclast differentiation and bone‐resorbing activity, as TRAP‐positive multinucleated cells represent functionally active osteoclasts (Ballanti et al. 1997; Solberg et al. 2014). In addition to TRAP staining, the assessment of resorption pits provides a direct measurement of the ability of osteoclasts to degrade mineralized substrates (Vesprey and Yang 2016). The organization of F‐actin into a peripheral ring structure is another characteristic feature of mature osteoclasts, reflecting cytoskeletal rearrangement required for the formation of a sealed resorption compartment (Han et al. 2019). In the present study, PF markedly decreased TRAP activity, limited resorption pit formation, and interfered with F‐actin ring organization. Collectively, these results indicate that PF suppresses not only osteoclast differentiation but also their functional bone‐resorptive capacity, suggesting its therapeutic relevance in conditions associated with excessive bone loss. These findings provide a functional basis for the subsequent analysis of molecular mechanisms underlying PF‐mediated inhibition of osteoclastogenesis.

To further elucidate the molecular mechanisms underlying these inhibitory effects, NFATc1 functions as a central transcriptional regulator in osteoclast differentiation. It governs the transcription of multiple osteoclast‐associated genes, including TRAP, OSCAR, CTK, and DC‐STAMP (Zhao et al. 2010). RANKL‐mediated signaling promotes NFATc1 activation, thereby initiating gene programs required for osteoclast maturation, cytoskeletal organization, and survival (Feng et al. 2019). Increased NFATc1 expression has been correlated with enhanced osteoclast activity and bone resorption, whereas the suppression of NFATc1 significantly limits osteoclast formation and activity, highlighting its critical role in bone remodeling and pathological bone loss (J. H. Kim and Kim 2014). The upstream of NFATc1, c‐Fos—an AP‐1 family transcription factor—acts as an early regulator in response to RANKL stimulation and contributes to NFATc1 induction (Matsuo et al. 2000). The importance of this regulatory cascade is supported by observations that c‐Fos deficiency results in impaired osteoclast development and osteopetrotic phenotypes (Grigoriadis et al. 1994). In the present study, PF reduced the protein expression of both c‐Fos and NFATc1, indicating interference with the principal transcriptional network governing osteoclastogenesis. Through the suppression of this regulatory axis, PF may consequently attenuate downstream gene expression required for osteoclast differentiation and bone‐resorptive function.

OSCAR facilitates osteoclastogenesis by activating NFATc1 and mediates both osteoclast fusion and functional activity, linking bone resorption with immune pathways (Goettsch et al. 2011). CTK, a cysteine protease, is critical for degrading type I collagen in the bone matrix, a process essential for bone turnover and remodeling (Janiszewski et al. 2023). The dysregulation of CTK has also been implicated in pathological conditions such as osteoporosis and metastatic bone diseases (Ishikawa et al. 2001). MMP‐9 contributes to the migration and fusion of osteoclast precursors and matrix degradation, playing a dual role in osteoclast adhesion and resorption activity (Zhu et al. 2020). ATP6v0d2, a component of the vacuolar H+‐ATPase proton pump, acidifies the resorption lacuna, enabling mineral dissolution, while also regulating cytoskeletal organization and precursor fusion (Wu et al. 2009). DC‐STAMP is indispensable for multinucleation, a critical process for forming mature osteoclasts required for effective bone resorption (Yagi et al. 2005). These genes collectively represent key functional mediators of osteoclast differentiation, fusion, and resorptive activity. In this study, PF treatment led to a marked reduction in the mRNA levels of OSCAR, CTK, MMP‐9, ATP6v0d2, and DC‐STAMP, genes that are closely associated with osteoclast differentiation, matrix degradation, and cell fusion. Collectively, these alterations indicate that the inhibitory effects of PF on bone resorption may involve coordinated modulation of osteoclast‐related gene networks, likely in association with decreased NFATc1 and c‐Fos signaling.

To extend the in vitro findings, the effects of PF were examined in an OVX rat model, a well‐established experimental system for postmenopausal osteoporosis (Liu et al. 2021). The removal of the ovaries induces estrogen deficiency, which accelerates osteoclast‐mediated bone resorption and disturbs the equilibrium between bone formation and resorption, ultimately resulting in trabecular bone deterioration. In this experimental setting, estradiol administration was included as a pharmacological reference to confirm the responsiveness and validity of the OVX model.

Micro‐CT analysis indicated that PF treatment effectively attenuated OVX‐induced trabecular bone loss (Liu et al. 2021). In particular, PF administration was associated with improvements in trabecular bone volume and thickness, along with reduced trabecular separation and increased trabecular number. These microstructural changes suggest that PF preserves trabecular bone architecture and mitigates estrogen deficiency–induced skeletal deterioration in vivo.

Serum TRAP, a marker of osteoclast activity, did not differ significantly between the Sham and OVX groups in the present study, suggesting that systemic TRAP levels were not markedly altered under our specific experimental conditions. Although PF treatment significantly reduced serum TRAP levels compared with the OVX group, this finding should be interpreted cautiously, as it may reflect a relative modulation of circulating TRAP rather than a definitive suppression of osteoclast activity, especially given the lack of a significant OVX‐induced increase. Although some studies have reported increased serum TRAP following ovariectomy, it is well documented that serum TRAP levels do not consistently correlate with local bone resorption depending on the experimental setting and sampling time (Guo et al. 2022). Given this, the anti‐resorptive effect of PF is more robustly supported by our in vitro osteoclastogenesis assays and micro‐CT‐based structural improvements than by serum TRAP measurements alone.

ALP, although commonly used as a marker of osteoblast activity, can also be elevated during pathological bone turnover characterized by excessive osteoclast activity and compensatory remodeling responses (Lee et al. 2022). In this study, OVX rats exhibited increased ALP levels, indicative of altered bone turnover. PF administration attenuated the OVX‐induced elevation of ALP, which may reflect the normalization of bone turnover. However, since total ALP includes contributions from both bone and liver, and bone‐specific ALP was not assessed, the interpretation of these findings should be made with caution. In addition, the observed reduction in ALP may also reflect a potential decrease in osteoblast activity, which cannot be excluded based on the present data. Therefore, the in vivo findings primarily reflect osteoclast‐related processes, and the overall interpretation of bone remodeling should be made with caution. Further studies including comprehensive bone remodeling analysis are required to clarify the effects of PF on overall bone remodeling.

Taken together, the present findings indicate that PF attenuates osteoclast differentiation and bone‐resorptive function, preserves trabecular microstructure, and contributes to the normalization of dysregulated bone turnover. The parallel effects observed in RANKL‐stimulated cells and the OVX rat model suggest potential translational relevance and support further exploration of PF in osteoporosis research. Because PF is widely used as a culinary spice, these findings may also support the concept that food‐derived phytochemicals can influence bone metabolism and contribute to dietary strategies for maintaining skeletal health. Nevertheless, several limitations should be acknowledged when interpreting these outcomes.

First, prior investigations have described the chemical profiles and biological activities of aqueous extracts of PF; however, the 100% ethanol extract examined in the present study was not comprehensively characterized at the constituent level. Consequently, the specific compounds contributing to the observed suppression of osteoclast differentiation cannot be conclusively determined. Ethanol extraction has been reported to preferentially enrich relatively lipophilic alkaloids and amide compounds, including piperine and related constituents, which are known to modulate inflammatory signaling and cellular differentiation pathways (Wan et al. 2023). In the present study, piperine was identified as a representative constituent of the PF extract, with a quantified content of 25.3 mg/g based on comparison with an authentic standard. However, the PF extract contains multiple constituents, and the observed biological effects cannot be attributed to piperine alone. The specific contribution of piperine to the observed effects could not be determined in the present study. Furthermore, as comprehensive chemical profiling of the extract was not performed, the characterization of PF remains limited, and therefore, the extract should not be considered a fully standardized product at this stage.

In contrast, aqueous extracts are generally enriched in more polar constituents and have often been applied in studies focusing on safety evaluation or general bone‐protective effects (Hoang et al. 2023). Therefore, while the use of a 100% ethanol extract in the present study may contribute to the inhibitory effects on osteoclast differentiation and bone resorption, the absence of comprehensive chemical profiling limits definitive conclusions regarding the precise active components involved. In addition, the current level of chemical characterization, based primarily on a single marker compound, is insufficient to support full standardization of the extract.

Second, the in vivo evaluation in the present study was limited to an ovariectomized osteoporosis model, which predominantly reflects postmenopausal bone loss associated with estrogen deficiency. Although this model is well established, other forms of osteoporosis, such as glucocorticoid‐induced or age‐related osteoporosis, were not examined. Therefore, the applicability of PF to broader osteoporotic conditions remains to be determined.

Third, although PF suppressed osteoclast differentiation and resorptive function, its influence on osteoblast‐mediated bone formation was not examined in the present study. Because bone remodeling depends on the dynamic interplay between osteoclasts and osteoblasts, key aspects of osteoblast function, including proliferation, differentiation, and mineralization, were not evaluated. Therefore, the evaluation of osteoblast‐related markers and functional parameters would be necessary to clarify the overall impact of PF on skeletal homeostasis.

Finally, although pharmacological comparisons between aqueous and ethanol extracts of PF would provide valuable insight, direct comparisons remain limited by differences in experimental systems. The present study employed RANKL‐induced RAW 264.7 cells, whereas previous studies using aqueous extracts relied on co‐culture models with osteocyte‐like MLO‐Y4 cells. In addition, the absence of long‐term in vivo studies restricts the assessment of PF's safety and efficacy over extended treatment periods. Accordingly, additional studies conducted under standardized experimental settings and extended treatment schedules are warranted to better characterize the sustained efficacy and broader therapeutic properties of PF.

## 5 Conclusion

In summary, ethanol‐extracted PF suppressed osteoclast differentiation and resorptive function and preserved trabecular bone microarchitecture in an ovariectomized osteoporosis model. PF administration also mitigated estrogen deficiency–associated bone loss. These findings suggest that PF may exert beneficial effects on bone metabolism, primarily through the inhibition of osteoclast‐related processes. However, given the limitations of the present study, including the lack of comprehensive evaluation of bone remodeling, further studies are required to clarify the effects of PF on overall bone remodeling in osteoporosis.

## Author Contributions

**Jin‐Ho Moon**: conceptualization, data curation, formal analysis, investigation, methodology, validation, writing – original draft, writing – review and editing. **Eun‐Young Kim**: conceptualization, investigation, methodology, validation, writing – review and editing, writing – original draft, data curation, formal analysis. **Sumin Lee**: formal analysis, investigation, methodology, visualization. **Won Jeong Shin**: formal analysis, investigation, methodology, visualization. **Seoung Jun Kwon**: formal analysis, investigation, methodology, visualization. **Youngwoo Nam**: investigation, visualization. **Youngjoo Sohn**: conceptualization, project administration, supervision, writing – review and editing. **Hyuk‐Sang Jung**: conceptualization, project administration, writing – review and editing, supervision.

## Funding

This work was supported by a grant from the Korea Health Technology R&D Project through the Korea Health Industry Development Institute (KHIDI), funded by the Ministry of Health and Welfare, Republic of Korea (grant number: HF21C0092).

## Conflicts of Interest

The authors declare no conflicts of interest.

## Supporting information



Supplementary table S1. List of materials and reagents used in this study.
Supplementary Table S2. Primer sequences for qPCR analysis.
Supplementary Table S3. Antibodies used for Western blot analysis.

## Data Availability

The data supporting the findings of this study are available from the corresponding author upon reasonable request.
