# Supplementary material for: Ethanol Extract of Piper longum L., a Culinary Spice, Suppresses Osteoclastogenesis and Protects Against Ovariectomy‐Induced Bone Loss
Source: J Food Sci. 2026 Jun 24;91(6):e71232. doi: 10.1111/1750-3841.71232 (PMC13292184; doi:10.1111/1750-3841.71232)
Supplement: Supplementary file 1 — Supplementary table S1. List of materials and reagents used in this study. Supplementary Table S2. Primer sequences for qPCR analysis. Supplementary Table S3. Antibodies used for Western blot analysis. [file JFDS-91-0-s001.docx]

**Supplementary table S1. List of materials and reagents used in this study.**

| **Materials and Reagents** | **Source** | **Cat No.** | **Location** |
| --- | --- | --- | --- |
| Receptor activator of nuclear factor κB ligand (RANKL) | PeproTech | 315-11C | Rocky Hill, NJ, USA |
| Piperis Fructus (PF) | Omniherb Co. | Batch No. D1909095159I | Uiseong, Gyeongsangbuk-do, Korea |
| RAW 264.7 cells | Korean Cell Line Bank (KCLB) | 40071 | Seoul, Korea |
| Dulbecco’s Modified Eagle Medium (DMEM) | Welgene | LM001-05 | Gyeongsan, Gyeongbuk, Korea |
| α-MEM | Gibco | 12571-063 | Grand Island, NY, USA |
| Fetal Bovine Serum (FBS) | SERANA | S-FBS-AU-015 | Brandenburg, Germany |
| Penicillin/Streptomycin (P/S) | Gibco | 15140-122 | Grand Island, NY, USA |
| Dulbecco’s Phosphate-Buffered Saline (DPBS) | Welgene | LB001-02 | Gyeongsan-si, Gyeongbuk, Korea |
| Cell Counting Kit-8 (CCK-8) | Dojindo Molecular Technologies | CK04 | Rockville, MD, USA |
| Tartrate-resistant acid phosphatase (TRAP) kit | Sigma-Aldrich | 387A | St. Louis, MO, USA |
| Osteo assay plates | Corning | 3987 | Corning, NY, USA |
| Bicinchoninic Acid (BCA) Protein Assay Kit | Takara Bio | T9300A | Otsu, Shiga, Japan |
| 17β-Estradiol (E2) | Sigma-Aldrich | E8875 | St. Louis, MO, USA |
| Acti-stain™ 488 Fluorescent Phalloidin | Cytoskeleton, Inc. | PHDG1 | Denver, CO, USA |
| 4′,6-Diamidino-2-phenylindole (DAPI) | Sigma-Aldrich | D9542 | St. Louis, MO, USA |
| RNAiso Plus | Takara Bio, Inc. | 9109 | Otsu, Shiga, Japan |
| SuperScript II Reverse Transcriptase kit | Invitrogen | 18064-014 | Carlsbad, CA, USA |
| KAPA Taq DNA Polymerase kit | Kapa Biosystems | KK3009 | Wilmington, MA, USA |
| Enhanced chemiluminescence (ECL) solution | Cytiva | RPN2106 | Marlborough, MA, USA |
| Protran™ NC Membranes 0.45μm | Cytiva | 10600002 | Marlborough, MA, USA |
| Protease inhibitors | Sigma-Aldrich | P8340 | St. Louis, MO, USA |
| Phosphatase inhibitors 2 | Sigma-Aldrich | P5726 | St. Louis, MO, USA |
| Phosphatase inhibitors 2 and 3 | Sigma-Aldrich | P0044 | St. Louis, MO, USA |
| Primers | Genotech | Custom | Daejeon, Korea |
| Peroxidase AffiniPure Goat Anti-Mouse IgG (H+L) | Jackson ImmunoResearch | 115-035-062 | West Grove, PA, USA |
| HRP-conjugated goat anti-rabbit IgG (H+L) | Jackson ImmunoResearch | 111-035-045 | West Grove, PA, USA |
| Anti-c-Fos antibody | Santa Cruz Biotechnology | sc-447 | Dallas, TX, USA |
| Anti-NFATc1 antibody | BD bioscience | BD-556602 | San Jose, CA, USA |
| Anti-β-actin antibody | Santa Cruz Biotechnology | sc-8432 | Dallas, TX, USA |

**Supplementary Table S2. Primer sequences for qPCR analysis.**

| **Target** | **Sequence (5'-3')** | **Length (bp)** | **Accession no.** |
| --- | --- | --- | --- |
| RANK | F: TGGCTACCACTGGAACTCAGAC | 107 | NM_009399.5 |
|  | R: TGCACACCGTATCCTTGTTGAG |  |  |
| TRAP | F: CACTCCCACCCTGAGATTTGT | 118 | NM_007388.3 |
|  | R: CATCGTCTGCACGGTTCTG |  |  |
| ATP6v0d2 | F: GACCCTGTGGCACTTTTTGT | 248 | NM_175406.3 |
|  | R: GCTTGCATTTGGGGAATCTA |  |  |
| DC-STAMP | F: TCCTCCATGAACAAACAGTTCCAA | 149 | NM_029422.4 |
|  | R: AGACGTGGTTTAGGAATGCAGCTC |  |  |
| MMP-9 | F: GGACCCGAAGCGGACATTG | 139 | NM_013599.5 |
|  | R: CGTCGTCGAAATGGGCATCT |  |  |
| CTK | F: GAAGAAGACTCACCAGAAGCAG | 102 | NM_007802.4 |
|  | R: TCCAGGTTATGGGCAGAGATT |  |  |
| OSCAR | F: GGAATGGTCCTCATCTCCTT | 125 | NM_175632.3 |
|  | R: TCCAGGCAGTCTCTTCAGTTT |  |  |
| NFATc1 | F: GCTTCACCCATTTGCTCCAG | 129 | NM_001164109.1 |
|  | R: ATGGTGTGGAAATACGGTTGGTC |  |  |
| c-Fos | F: CGGGTTTCAACGCCGACTA | 165 | NM_010234.3 |
|  | R: TGGCACTAGAGACGGACAGAT |  |  |
| GAPDH | F: GCACAGTCAAGGCCGAGAAT | 151 | NM_001411842.1 |
|  | R: GCCTTCTCCATGGTGGTGAA |  |  |
| Abbreviations: RANK, receptor activator of nuclear factor-κB; TRAP, tartrate-resistant acid phosphatase; ATP6v0d2, ATPase H+ transporter V0 subunit d2; DC-STAMP, dendritic cell-specific transmembrane protein; MMP-9, matrix metallopeptidase-9; CTK, Cathepsin K; OSCAR, osteoclast-associated receptor; NFATc1, nuclear factor of activated T cells cytoplasmic 1; c-Fos, cellular Fos proto-oncogene; GAPDH, glyceraldehyde-3-phosphate dehydrogenase. | | | |

**Supplementary Table S3. Antibodies used for Western blot analysis.**

| **Application** | **Antigen** | **Origin** | **Antibody dilution** | **Supplier** | **Cat. no.** |
| --- | --- | --- | --- | --- | --- |
| Primary  antibodies | NFATc1 | mouse | 1:1,000 | BD Biosciences | 556602 |
|  | c-Fos | mouse | 1:1,000 | Santa Cruz | sc-447 |
|  | Actin | mouse | 1:1,000 | Santa Cruz | 8432 |
| Secondary  antibodies | Peroxidase AffiniPure® Goat Anti-Mouse IgG | Goat | 1:10,000 | Jackson | 111-035-045 |
| Abbreviations: NFATc1, nuclear factor of activated T cells cytoplasmic 1; c-Fos, cellular Fos proto-oncogene. | | | | | |
